# The Fabrication and *in vitro* Evaluation of Retinoic Acid-Loaded Electrospun Composite Biomaterials for Tracheal Tissue Regeneration

**DOI:** 10.3389/fbioe.2020.00190

**Published:** 2020-03-20

**Authors:** Cian O'Leary, Luis Soriano, Aidan Fagan-Murphy, Ivana Ivankovic, Brenton Cavanagh, Fergal J. O'Brien, Sally-Ann Cryan

**Affiliations:** ^1^School of Pharmacy and Biomolecular Sciences, Royal College of Surgeons in Ireland, Dublin, Ireland; ^2^Tissue Engineering Research Group, Department of Anatomy and Regenerative Medicine, Royal College of Surgeons in Ireland, Dublin, Ireland; ^3^SFI Advanced Materials and Bioengineering Research (AMBER) Center, Royal College of Surgeons in Ireland and Trinity College Dublin, Dublin, Ireland; ^4^SFI Center for Research in Medical Devices (CÚRAM), Royal College of Surgeons in Ireland, Dublin, Ireland; ^5^Cellular and Molecular Imaging Core, Royal College of Surgeons in Ireland, Dublin, Ireland; ^6^Trinity Center for Biomedical Engineering, Trinity College Dublin, Dublin, Ireland

**Keywords:** tracheal regeneration, retinoic acid, polycaprolactone, chitosan, biomaterials, mucociliary epithelium, electrospinning, composite

## Abstract

Although relatively rare, major trauma to the tracheal region of the airways poses a significant clinical challenge with few effective treatments. Bioengineering and regenerative medicine strategies have the potential to create biocompatible, implantable biomaterial scaffolds, with the capacity to restore lost tissue with functional neo-trachea. The main goal of this study was to develop a nanofibrous polycaprolactone-chitosan (PCL-Chitosan) scaffold loaded with a signaling molecule, all-*trans* retinoic acid (atRA), as a novel biomaterial approach for tracheal tissue engineering. Using the Spraybase® electrospinning platform, polymer concentration, solvent selection, and instrument parameters were optimized to yield a co-polymer with nanofibers of 181–197 nm in diameter that mimicked tracheobronchial tissue architecture. Thereafter, scaffolds were assessed for their biocompatibility and capacity to induce mucociliary functionalization using the Calu-3 cell line. PCL-Chitosan scaffolds were found to be biocompatible in nature and support Calu-3 cell viability over a 14 day time period. Additionally, the inclusion of atRA did not compromise Calu-3 cell viability, while still achieving an efficient encapsulation of the signaling molecule over a range of atRA concentrations. atRA release from scaffolds led to an increase in mucociliary gene expression at high scaffold loading doses, with augmented MUC5AC and FOXJ1 detected by RT-PCR. Overall, this scaffold integrates a synthetic polymer that has been used in human tracheal stents, a natural polymer generally regarded as safe (GRAS), and a drug with decades of use in patients. Coupled with the scalable nature of electrospinning as a fabrication method, all of these characteristics make the biomaterial outlined in this study amenable as an implantable device for an unmet clinical need in tracheal replacement.

## Introduction

Although relatively rare, major trauma to the tracheal region of the airways poses a significant clinical challenge with few effective treatments (Prokakis et al., 2014; Etienne et al., 2018). The current standard of care for extensive segmental damage in the trachea entails surgical resection of the lesion and end-to-end anastomosis that reconnects the tubular pathway for oxygen conduction to the lungs; however, this approach is not possible in cases where over half of the tracheal tissue length is damaged in adults, or one third in children (Grillo, 2002). On the other hand, replacement prostheses are significantly hindered by complications including a lack of implant epithelialization, device dislodgement, or for pediatric patients with congenital tracheal stenosis, limited scope for implant integration as the native trachea grows (Walles, 2011; Elliott et al., 2012; Hamilton et al., 2015). As such, bioengineering and regenerative medicine strategies continue to be investigated as a platform to create biocompatible, implantable biomaterial scaffolds, with the capacity to restore lost tissue and subsequently resorb *in situ* and be replaced by viable, functional neo-trachea (O'Leary et al., 2015). However, albeit a promising next-generation approach, the most high-profile bioengineered tracheal constructs to date have experienced significant drawbacks, including the need for donor supply and mechanical collapse with decellularized tissue or insufficient biocompatibility and cell growth on synthetic biomaterials (Vogel, 2013; Macchiarini, 2016). Thus, the optimal biomaterial for tracheal tissue engineering still remains to be determined for a meaningful clinical solution to long segmental trauma.

In the pursuit of an optimal biomaterial, it is important to consider the anatomical and histological characteristics of the trachea. Unlike most other tissue engineering applications involving internal organs such as bone, heart, and nerve, the trachea is located in a non-sterile environment at an external interface in the airway lumen; consequently, it is paramount for the biomaterial to exhibit epithelial cell biocompatibility. Bioengineered implants with inadequate epithelial cell coverage are predisposed to bacterial colonization, inflammatory responses, and resultant secretory mucus accumulation or stenosis that can block the airways (Zhang et al., 2015). Accordingly, recent work by our group has focused on the development of natural polymeric bioengineered scaffolds that support the growth and differentiation of a functional tracheobronchial epithelium, complete with epithelial confluence and mucociliary activity (O'Leary et al., 2016). These scaffolds have been subsequently modified for tracheal tissue regeneration through the incorporation of all-*trans* retinoic acid (atRA), a bioactive molecule that enhanced mucociliary epithelial functionalization (O'Leary et al., 2017), highlighting the potential for atRA-eluting biomaterials as future implantable tracheal constructs. However, these scaffolds suffered from a principal drawback- namely, the inefficient encapsulation of the hydrophobic atRA molecule into the more hydrophilic, collagen-based material. Alternatively, the use of hydrophobic polymers within the scaffold can improve the incorporation of atRA (Cirpanli et al., 2005; Puppi et al., 2010; Almouazen et al., 2012; Jeong et al., 2013), prompting further investigation as biomaterials for tracheal repair. To this end, we outline herein the development and characterization of a nanofibrous scaffold composed of a synthetic and natural polymer blend and loaded with atRA, as a novel biomaterial approach for tracheal tissue engineering.

Polycaprolactone (PCL) is a biocompatible, hydrophobic, and resorbable polymer with the potential to incorporate atRA with greater efficiency. It is an aliphatic polymer with a long residence time *in vivo* and exhibits pseudoplastic properties that resemble mechanical behavior of the body's extracellular matrix (ECM), making it an attractive material for regenerating organs with greater mechanical loads (Grosvenor and Staniforth, 1996; Woodruff and Hutmacher, 2010). Moreover, PCL can be used for amenable and scalable scaffold fabrication with a variety of methods that is not possible for less robust natural polymers; in particular, its capacity to be produced as a fibrous material via electrospinning is a distinct advantage, given the nanofibrous nature of tracheal tissue (Harrington et al., 2014; Bridge et al., 2015). For respiratory applications, PCL has been successfully used as tracheobronchial splints to maintain airway patency, in addition to reinforcing silicone tubes as analogs of cartilaginous tracheal rings (Zopf et al., 2013, 2014; Tsao et al., 2014; Hollister et al., 2015). In these seminal studies, PCL was predicted to fully degrade in the tracheal region after 3 years by surface erosion, as observed in other implants. However, the polymer has generally performed best in the tracheal replacement setting in composite form with natural biomaterials, including collagen (Lin et al., 2008, 2009), fibrin (Chang et al., 2014), or gelatin and decorin (Hinderer et al., 2012). To this end, our study proposed to combine PCL with a naturally-derived material, chitosan that could also be electrospun to reduce multiple manufacture steps and had demonstrated potential as a substrate for respiratory epithelium (Risbud et al., 2001; Huang et al., 2009; Qasim et al., 2018).

Therefore, the main goal of this study was to develop a nanofibrous polycaprolactone-chitosan (PCL-Chitosan) scaffold loaded with atRA as a novel bioactive scaffold for tracheal tissue engineering. In the pursuit of this main objective, three specific objectives were established: to initially electrospin the PCL-Chitosan polymer blend and validate its biocompatibility with respiratory epithelial cells, to successfully incorporate atRA into the PCL-Chitosan scaffold with high encapsulation efficiency, and finally, to evaluate the ability of the atRA-loaded scaffold to support epithelial cell growth and functionalization.

## Materials and Methods

### Materials

Unless specified in the text, all materials and reagents were supplied by Sigma-Aldrich (Arklow, Ireland).

### Nanofibrous Polycaprolactone-Chitosan (PCL-Chitosan) Scaffold Development

#### Preparation of Polymer Solutions

PCL (M_w_ 80,000 g/mol) solutions for electrospinning were prepared by polymer dissolution in 87.5% (v/v) chloroform and 12.5% (v/v) methanol with sonication to provide preparations of 5–20% (w/v). For composite PCL-Chitosan solutions, chitosan was initially dissolved overnight in 90% (v/v) acetic acid at 60°C to provide a 0.5% (w/v) solution, followed by dropwise addition of an equal volume of PCL solution with stirring prior to electrospinning.

#### Electrospinning

Scaffold fabrication was performed with a Spraybase® electrospinning platform (AVECTAS, Maynooth, Ireland). This setup consisted of a grounded collector plate, a high voltage (HV) emitter, syringe pump (Spraybase® SyringePumpPro), and a digital camera (PointGrey, Chameleon) for visualization of the Taylor cone. Each polymer solution was loaded into a 10 ml syringe and pumped at rates of 5–100 μL/min at a potential of 7–10 kV, depending on the specific solution being electrospun (Supplmentary Table 1). All scaffolds were manufactured at room temperature and at 20–40% humidity.

#### Scaffold Ultrastructure

The ultrastructure and fiber diameter of scaffolds were examined using scanning electron microscopy (SEM). Scaffold samples were mounted onto aluminum stubs with either double-sided adhesive tabs or conductive graphite adhesive and subsequently sputter-coated with a gold following this the sample was splutter coated with gold-palladium. Images were captured with a Carl Zeiss Ultra SEM (Cambridge, UK).

### All-*trans* Retinoic Acid (atRA)-Loaded Scaffold Manufacture and Characterization

#### Scaffold Fabrication

atRA was incorporated into PCL-Chitosan scaffolds at a range of concentrations to evaluate the dose-responsive differentiation of epithelial cells into a functional tracheobronchial epithelium. To this end, atRA was dissolved into 10% PCL-0.5% chitosan polymer solutions (section Preparation of Polymer Solutions) to provide concentrations of 0.1, 1, or 10 μg/mg, expressed as atRA quantity per mg of total scaffold.

#### Loading Capacity and Encapsulation Efficiency of atRA

The post-electrospinning quantity of atRA loaded into scaffolds was assessed by biomaterial dissolution and UV-Vis spectroscopy. Scaffold samples were dissolved by acetone in combination with sonication for 30–60 min in order to extract the loaded drug. The solution was then filtered through a 45 μm filter followed by absorbance measurement at 350 nm and atRA quantification, with reference to a standard calibration curve.

### Cell Culture

#### Cell Selection and Culture Media

The Calu-3 bronchial epithelial cell line (ATCC, Middlesex, UK) was cultured in a 1:1 mixture of Dulbecco's modified Eagle's medium and Ham's F12 medium supplemented with 10% fetal bovine serum (Biosera, Ringmer, UK), 2 mM L-glutamine, 14 mM sodium bicarbonate and 100 U/ml penicillin/streptomycin. Cells were used between passages 20–40. Unless otherwise stated, all cell culture and incubation steps were at 37°C and 5% CO_2_ in a humidified atmosphere.

#### Cell Culture on Scaffolds

Calu-3 cells were cultured under air-liquid interface (ALI) conditions on scaffolds for 14 days in order to assess the biomaterial's ability to support respiratory epithelial growth and differentiation. 15.6 mm-diameter scaffolds were fastened in a customized ALI culture system using plastic frames of Snapwell® inserts (Corning, NY, USA) as previously described (O'Leary et al., 2016). Calu-3 cells were seeded at 5 × 10^5^ cells/cm^2^ and cultured for 3 days, submerged with medium in the apical and basolateral compartment. Thereafter, the medium was removed from the apical compartment to introduce an ALI and the cells were fed via the basolateral compartment for a further 11 days, with medium replaced every 2–3 days.

Bilayered collagen-hyaluronate scaffolds previously optimized for respiratory epithelial cell growth (O'Leary et al., 2016) were used as a positive control for initial viability assessment of PCL and PCL-Chitosan biomaterials. For atRA studies, drug-free scaffolds in the absence or presence of supplementation with 0.3 μg/ml atRA (equivalent to 1 × 10^−7^ M) were included as negative or positive controls, respectively.

### *In vitro* Analysis

#### Biocompatibility

Cell viability was assessed by a combination of qualitative and quantitative methods. Firstly, cell-seeded samples were washed in PBS and incubated with alamarBlue® (Thermo Fisher, Dublin, Ireland) to assay cell viability by reduction of resazurin. Thereafter, a Live/Dead® cell imaging kit (Thermo Fisher) was used to visualize viable and non-viable epithelial cells. All kits were used according to the manufacturer's protocol.

#### Mucin Expression

Immunofluorescent staining of MUC5AC was carried out to assess the effect of atRA-loading on the induction and maintenance of mucin secretion, a phenotypic marker of tracheobronchial epithelium. Cell-seeded samples were stained as previously described (O'Leary et al., 2016, 2017), with mouse anti-MUC5AC antibody (Abcam ab24070, Cambridge, UK) incubated at a dilution of 1/100 for 2 h at room temperature. Images were captured and analyzed using an Axio Examiner.Z1 confocal microscope (Carl Zeiss).

#### Ciliation

Cell-seeded samples were analyzed by TEM to identify the presence of cilia on Calu-3 epithelial cells and to observe cell morphology. Scaffold and cell insert samples were washed in PBS and fixed in 10% neutral buffered formalin for 20 min prior to treatment. The samples were then stained with 1% osmium tetroxide for 1 h, followed by dehydration using descending grades of methanol. They were subsequently immersed in a 1:1 methanol:London resin white and finally in pure London resin white for 1 h at room temperature. The samples were then embedded and ultrathin sections were generated using an EM UC6 ultramicrotome (Leica) and mounted on copper grids prior to examination in a Hitachi H-7650 electron microscope operating at 100 kV.

#### Expression of Genetic Markers of Tracheobronchial Differentiation

The ability of the atRA-loaded scaffolds to support tracheobronchial epithelial differentiation was further analyzed by quantitative relative gene expression of MUC5AC and FOXJ1 as genetic markers for mucus production and ciliation, respectively. RNA was isolated from cell lysates using an RNeasy kit (Qiagen, Crawley, UK) and quantified by absorption spectroscopy at 260 nm. 200 ng of total RNA was reverse transcribed to cDNA using a QuantiTect reverse transcription kit (Qiagen). RT-polymerase chain reactions were run on a 7,500 real-time PCR System (Applied Biosystems, UK) using a QuantiTect SYBR Green PCR Kit (Qiagen) with QuantiTect primers (Qiagen). The expression of mRNA was calculated by the delta-delta Ct (2^−ΔΔ*Ct*^) method relative to the housekeeping gene 18S (Livak and Schmittgen, 2001), with gene expression compared to that on atRA-free scaffolds.

### Data Analysis

Analysis of microscopy images was performed using the Fiji (Fiji is just ImageJ) processing software. Quantitative data obtained were analyzed using Microsoft Excel and GraphPad Prism 7.0 Software. In cases of analysis between two groups, statistical difference was assessed by two-tailed Student *t*-test. For multiple groups, statistical difference between groups was assessed by 1-way or 2-way ANOVA, as appropriate. Bonferroni *post hoc* analysis was performed in all ANOVA assessments.

## Results

### Nanofibrous Polycaprolactone-Chitosan (PCL-Chitosan) Scaffold Development

A combination of PCL and chitosan were electrospun together to produce a scaffolds that combined the advantages of PCL's nanofibrous architecture with chitosan's biocompatibility as a substrate for respiratory epithelium. Following the production of PCL scaffolds with a series of heterogeneously-distributed and large-diameter fiber production (Supplementary Table 1), a change from an 18G needle to a narrower-diameter 22G in the HV emitter coupled with a reduction in the applied potential to provide a flow rate of 10 μL/min both improved the manufacture of scaffolds with reproducible fiber diameters in the dimensional range of tracheal tissue (Harrington et al., 2014; Bridge et al., 2015).

**Figure 1:**
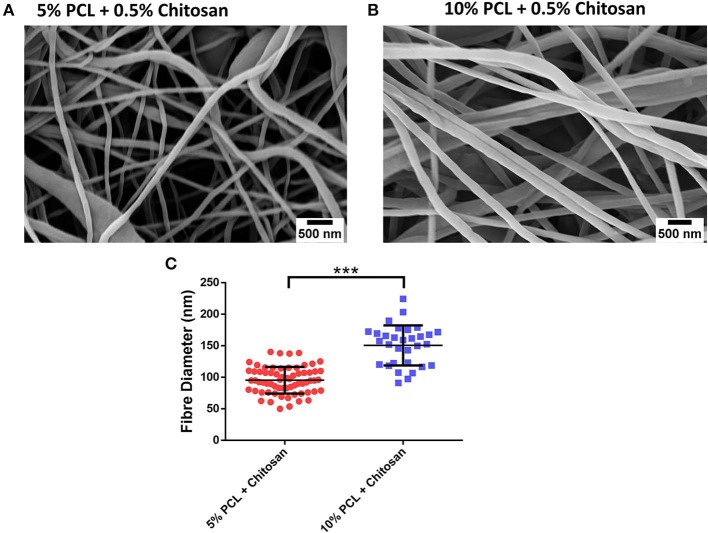
Fabrication of polycaprolactone (PCL)-chitosan scaffolds. Representative images of **(A)** 5%/0.5% and **(B)** 10%/0.5% PCL-chitosan formulations. **(C)** Mean diameter of electrospun fibers. Results displayed as mean ± SD. *n* ≥ 31 fibers analyzed across a minimum of two scaffold batches. ***p < 0.001.

Using these data as a guide, chitosan was incorporated into the PCL feed solution to spin fibers composed of a polymer blend of PCL and chitosan (Figure 1). At concentrations of 5 and 10% PCL with 0.5% chitosan, homogenous fibers with mean diameters of 95 and 151 nm were formed, respectively (Figures 1A,B), resulting in a statistically-significant increase in diameter with increased PCL concentrations (Figure 1C; *p* < 0.001). Subsequent increases in PCL concentration failed to yield nanofibers, however, as a result of the solution drying too quickly in the electrospinning process (Table 1), necessitating a compromise between PCL concentration in combination with chitosan to produce fibers that could be effectively loaded with a high dose of signaling molecules without significantly increasing fiber diameter.

**Table 1:** Polycaprolactone-chitosan (PCL-Chit) nanofiber production.

**Formulation (% w/v)**	**Needle gauge**	**Flow rate (μL/min)**	**Fiber diameter (nm)**
PCL 5%	22G	10 25	88 ± 46 57 ± 33
PCL 10%	22G	10 25	344 ± 92 357 ± 262
PCL 5% + Chitosan	22G	10 25	95 ± 18 87 ± 31
PCL 10% + Chitosan	22G	10 25	151 ± 35 102 ± 21
PCL 15% + Chitosan	22G	10 25	Solution dried too quickly at all flow rates to be usable

*Chitosan was incorporated into PCL scaffolds at a concentration of 0.5%. Results presented as mean diameter ± SD. n ≥ 31 fibers analyzed across a minimum of two scaffold batches*.

### Biocompatibility of PCL-Chitosan Scaffolds

**Figure 2:**
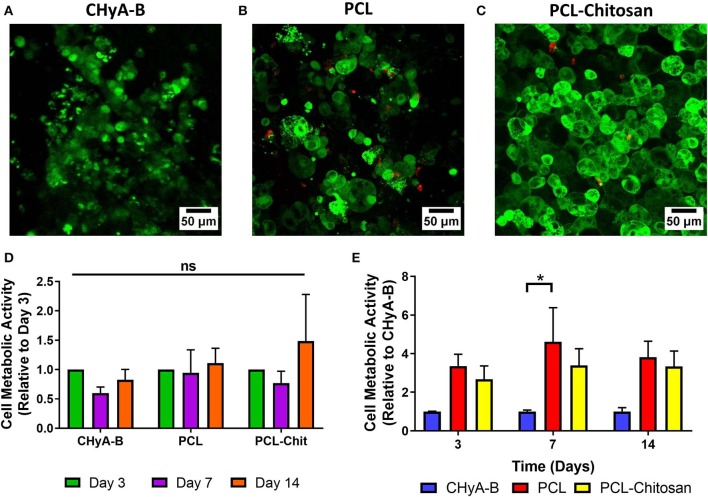
Biocompatibility of 10% polycaprolactone−0.5% chitosan (PCL-Chit) scaffolds. Representative live/dead images of Calu-3 cells cultured on **(A)** bilayered collagen-hyaluronate (CHyA-B), **(B)** polycaprolactone (PCL), or **(C)** PCL-chitosan (PCL-Chitosan) scaffolds for 14 days. Live cells are shown in green and dead cells appear as red. **(D,E)** Analysis of cell metabolic activity by alamarBlue® reduction. Results displayed as mean ± SEM, with relative metabolic activity expressed as **(D)** a function of time for each group or **(E)** as a function of activity on CHyA-B scaffolds at each individual time point. *n* = 4 biological replicates performed in duplicate. *p < 0.05.

The ability of the 10% PCL-0.5% chitosan scaffolds to support Calu-3 epithelial cells was determined by analysis of reduction of alamarBlue® and Live/Dead® cell imaging (Figure 2) and compared to Calu-3 cells cultured on the CHyA-B scaffold control group (Figure 2A). Overall, the PCL and PCL-chitosan scaffolds were found to be biocompatible in nature and support Calu-3 cell viability across a 14 day time period. Although cell imaging indicated the presence of some dead cells on both scaffolds (Figures 2B,C), this was negligible in comparison to the degree of green fluorescence for live cells observed on electrospun scaffolds. Moreover, analysis of alamarBlue® reduction demonstrated that, relative to cells on CHyA-B scaffolds, Calu-3 cells exhibited a non-significant increase in metabolic activity, with the exception of the PCL scaffold group on day 7 (Figures 2D,E). Additionally, cells were observed to spread more across the PCL-Chitosan biomaterial than on the PCL alone biomaterial. Taken together, these results indicated that the electrospun scaffolds were capable of supporting the growth of respiratory epithelial cells at an ALI and were therefore suitable to use for atRA incorporation on the basis of their biocompatibility in addition to their nanofibrous architecture.

### Nanofibrous All-*trans* Retinoic Acid (atRA)-Loaded Scaffold Development

**Figure 3:**
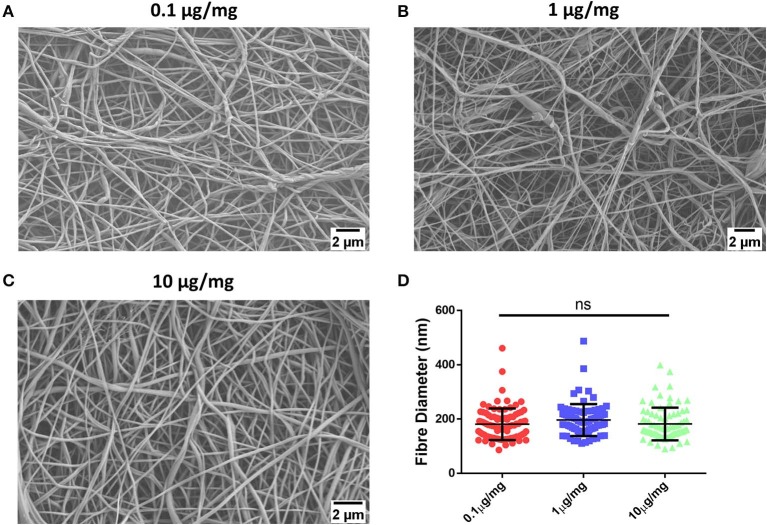
Incorporation of retinoic acid (atRA) into 10% polycaprolactone−0.5% chitosan scaffolds. Representative images of **(A)** 0.1 μg, **(B)** 1 μg, or **(C)** 10 μg atRA per mg of scaffold. **(D)** Mean diameter of electrospun fibers. Results displayed as mean ± SD. *n* ≥ 31 fibers analyzed across a minimum of two scaffold batches. ^ns^*p* > 0.05.

Having successfully fabricated the PCL-Chitosan nanofibres and validated their biocompatibility, our second objective sought to effectively incorporate the signaling molecule atRA into the scaffold. A range of atRA doses were loaded into the 10% PCL-0.5% Chitosan polymer blends prior to electrospinning; 0.1, 1, and 10 μg/mg. The atRA-loaded scaffolds were found to retain a homogenous nanofibrous structure (Figures 3A–C). Additionally, atRA concentration had no significant effect on fiber diameter (Figure 3D; *p* > 0.05), with diameters of 181, 197, and 182 nm for the 0.1, 1, and 10 μg/mg scaffolds respectively. In summary, the atRA-loaded PCL-Chitosan scaffolds maintained the desirable nano-architecture of the unloaded PCL-Chitosan scaffold.

### Loading Capacity and Encapsulation Efficiency

The quantity of atRA loaded into scaffolds post-electrospinning was assessed by dissolution of the fibers and quantification of atRA using UV-Vis spectroscopy. Spectroscopic analysis confirmed that a high level of atRA encapsulation of approximately 77–89% for each of the loading doses used i.e., 0.1–10 μg/mg (Table 2). Moreover, a proportional increase in loading capacity was seen for the different loading doses, with an almost tenfold increase in atRA loading capacity seen between the 0.1 and 1 μg/mg starting doses from 0.08 to 0.72 and over a 100-fold increase in atRA loading capacity between 0.1 and 10 μg/mg starting doses. This demonstrated both the reproducibility in the fabrication method and confirmed the PCL-Chit scaffold's capacity for high atRA loading.

**Table 2:** Loading capacity & Encapsulation efficiency of retinoic acid (atRA) loading into polycaprolactone-chitosan scaffolds.

**[atRA] Loading (μg/mg)**	**Yield**	
	**[atRA] (μg/mg)**	**EE (%)**
0.1	0.08 ± 0.01	76.5 ± 13.4
1	0.72 ± 0.21	72.3 ± 20.6
10	8.88 ± 1.33	88.9 ± 13.3

*atRA was loaded at concentrations of 0.1 μg, 1 μg, or 10 μg atRA per mg of scaffold. Results displayed as mean ± SD. n = 3 batches performed in triplicate*.

### Biocompatibility of atRA-Loaded PCL-Chitosan Scaffolds

**Figure 4:**
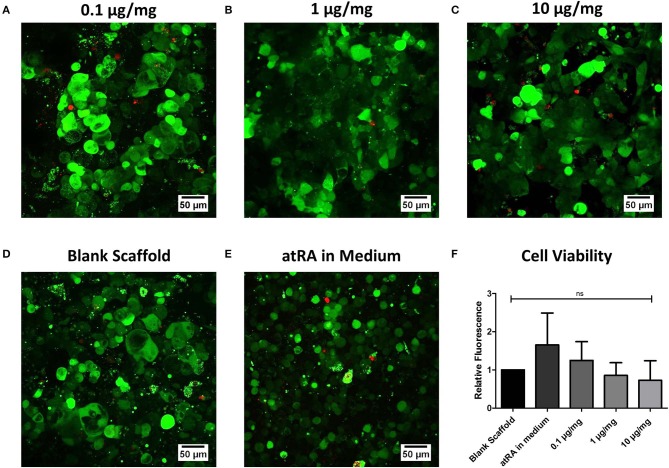
Biocompatibility of retinoic acid-loaded 10% polycaprolactone−0.5% chitosan scaffolds. Representative live/dead images of Calu-3 cells cultured on **(A)** 0.1 μg/mg, **(B)** 1 μg/mg, or **(C)** 10 μg/mg scaffolds for 14 days. Cells were also grown on drug-free blank scaffolds in the **(D)** absence or **(E)** presence of 0.3 μg/ml atRA in medium. Live cells are shown in green and dead cells appear as red. *n* = 4 biological replicates. **(F)** Analysis of cell metabolic activity by alamarBlue® reduction. Results displayed as mean ± SEM with expression relative to blank scaffolds. *n* = 3 biological replicates performed in duplicate.

Respiratory epithelial cell viability on atRA-loaded scaffolds was investigated as part of the biocompatibility assessment for the biomaterial (Figure 4). Overall, the inclusion of atRA did not compromise Calu-3 cell viability (Figures 4A–C), with similar visualization of live green cells observed to that of atRA-free PCL-Chit scaffolds (Figure 4D). Analysis of cell activity by alamarBlue® reduction confirmed this finding, although a non-significant trend toward reduced metabolic activity with higher atRA concentrations was recorded (Figure 4F). These studies confirmed that the atRA-loaded scaffolds were biocompatible substrates for respiratory epithelial cell growth, warranting further *in vitro* assessment of epithelial behavior.

### Mucin Expression as a Marker of Tracheobronchial Phenotype

**Figure 5:**
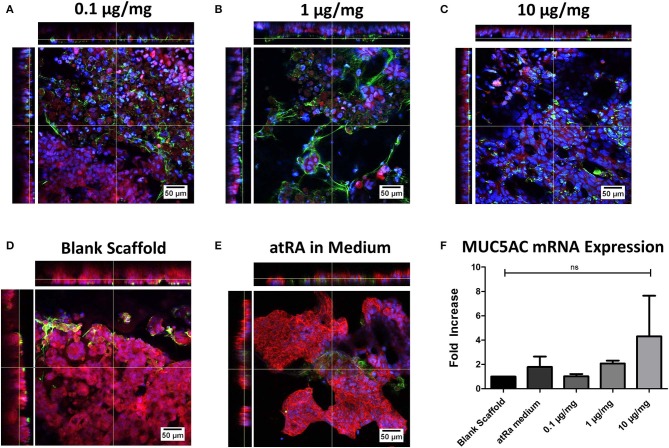
Calu-3 cell mucin expression on retinoic acid-loaded 10% polycaprolactone−0.5% chitosan scaffolds. Representative images display apical MUC5AC glycoprotein secretion (green) on **(A)** 0.1 μg/mg, **(B)** 1 μg/mg, or **(C)** 10 μg/mg scaffolds at 14 days. Cells were also grown on drug-free blank scaffolds in the **(D)** absence or **(E)** presence of 0.3 μg/ml atRA in medium. Samples were counterstained for nuclei (blue) and F-actin (red). *n* = 2 biological replicates. **(F)** Relative MUC5AC mRNA expression of cells. Results displayed as mean ± SEM with expression relative to blank scaffolds. *n* = 3 biological replicates performed in duplicate.

Immunofluorescent staining of MUC5AC glycoprotein and expression of the related MUC5AC gene was carried out to assess the effect of atRA-loading on the induction and maintenance of mucin secretion, a phenotypic marker of tracheobronchial epithelium (Figure 5). Although apical secretions of MUC5AC were visualized in all groups (Figures 5A–E), a greater degree of mucin expression was seen in the atRA-loaded scaffold groups (Figures 5A–C), with a more diffuse spreading pattern observed in the 1 μg/mg group (Figure 5B). Interestingly, confocal imaging revealed that all atRA-loaded scaffolds exhibited a greater extent of MUC5AC expression than that observed in atRA-supplemented medium (Figure 5E). Subsequent analysis of MUC5AC gene expression found in spite of the increase in glycoprotein expression, no statistically-significant relative increase in MUC5AC mRNA expression was detected for the cells grown on the atRA-loaded scaffolds at the same time point of 14 days (Figure 5F). It should be noted that an approximate 2-fold increase in MUC5AC mRNA expression was seen for cells cultured in the control atRA medium group and on the 1 μg/mg atRA-loaded scaffold group and an almost 4-fold increase was seen for cells cultured on the 10 μg/mg scaffold, albeit with high variability between replicate samples. Taken together, these data indicated that the mid-range of atRA loading i.e., 1μg/mg into PCL-Chit scaffolds stimulated an increase in mucin expression.

### Ciliation as a Marker of Tracheobronchial Phenotype

**Figure 6:**
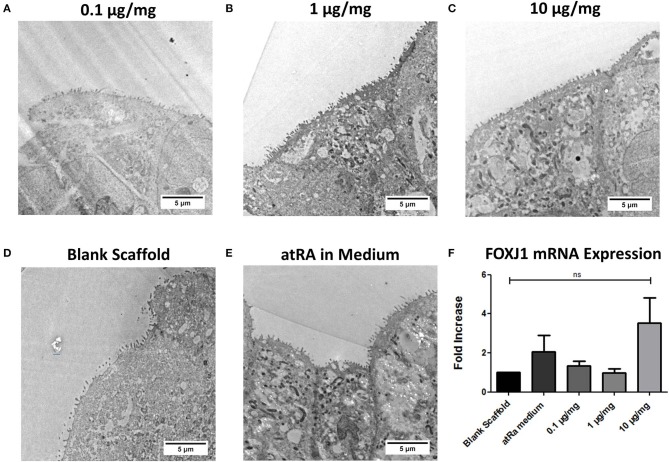
Calu-3 cell ciliation on retinoic acid-loaded 10% polycaprolactone−0.5% chitosan scaffolds. Representative images display cellular extensions from the apical surface of cells on **(A)** 0.1 μg/mg, **(B)** 1 μg/mg, or **(C)** 10 μg/mg scaffolds at 14 days. Cells were also grown on drug-free blank scaffolds in the **(D)** absence or **(E)** presence of 0.3 μg/ml atRA in medium. *n* = 1 biological replicate. **(F)** Relative FOXJ1 mRNA expression of cells. Results displayed as mean ± SEM with expression relative to blank scaffolds. *n* = 3 biological replicates.

TEM analysis of Calu-3 seeded (10% PCL-0.5% Chitosan) scaffolds and determination of expression of the FOXJ1 gene were carried out to assess the effect of atRA-loading on the development and maintenance of epithelial cilia, another central phenotypic marker of tracheobronchial epithelium (Figure 6). atRA did not induce any visible maturation or elongation of motile cilia (Figures 6A–E). In analysis of FOXJ1 mRNA expression, no statistically significant increases were found for cells grown on the atRA-loaded materials. However, an approximate 2-fold increase in FOXJ1 mRNA expression was seen for cells cultured in the control atRA medium group and an almost 4-fold increase, albeit variable, was seen for cells cultured on the 10 μg/mg scaffold (Figure 6F).

## Discussion

In the pursuit of an optimal biomaterial with the potential to regenerate lost tracheal tissue in large airway trauma, the objective of this study was to develop a nanofibrous polycaprolactone-chitosan (PCL-Chitosan) scaffold loaded with all-*trans* retinoic acid (atRA) as a novel bioactive material scaffold for tracheal tissue engineering. Specifically, we aimed to electrospin the PCL-Chitosan polymer blend, validate the biocompatibility of the fibers produced, incorporate atRA into the PCL-Chitosan scaffold, and finally, to evaluate the ability of the atRA-loaded scaffold to support cell growth and functionalization. Our results have led to the refinement of an electrospinning process to successfully fabricate a nanofibrous biomaterial scaffold composed of synthetic and natural polymers that can be effectively loaded with a small bioactive molecule, atRA. Moreover, this scaffold is biocompatible in nature and exhibits capacity to support respiratory epithelial functionality. Although further *in vitro* analysis with primary cells and *in vivo* examination is warranted to fully validate claims of scaffold integration and ciliation, this study has nonetheless demonstrated the feasibility of the manufacture of am atRA-loaded scaffold for tracheal regeneration.

Tracheal ECM is composed primarily of fibrillary collagens and elastin that adopt a compact, fibrous, submucosal base for the pseudostratified epithelium coating the luminal walls (Dunsmore and Rannels, 1996). In seminal studies, PCL has been predicted to fully degrade in the tracheal region after 3 years by surface erosion, as observed in other implants (Zopf et al., 2013, 2014; Tsao et al., 2014; Hollister et al., 2015). Having tested a range of solvents of that could successfully dissolve and maintain PCL and chitosan both in solution without precipitation prior to electrospinning, a process involving dissolution of chitosan in acetic acid followed by dropwise addition of an equal volume of PCL solution in chloroform and methanol. Microscopic analysis of decellularized tracheal ECM has revealed that these fibers are approximately 200–250 nm in diameter (Harrington et al., 2014; Bridge et al., 2015), setting the benchmark for the biomimetic PCL-based scaffold in this study. Once all three core components of the material were brought together following a stepwise optimization of electrospinning parameters and polymer stock concentrations, atRA-loaded PCL-Chitosan scaffolds were reproducibly fabricated that recapitulated tracheal nanofiber dimensions for a range of drug concentrations. While future extensive analysis of the co-polymer's macromolecular structure using Fourier transform infrared spectroscopy or x-ray diffraction could provide further insight, the overall presence of such a physiologically-representative architecture is considered a crucial determinant in facilitating epithelial cell confluence and deposition of their own basement membrane (Zhang et al., 2015; Karsdal et al., 2017), and as such, our scaffolds exhibited favorable structure as a substrate for respiratory epithelium.

Coupled with the capability of resembling tissue structure, the biomaterial scaffold must also be fundamentally biocompatible for the pertinent cells in order to succeed as an implant for tracheal regeneration. PCL scaffolds have been evaluated for epithelial cell attachment and growth for several epithelial cell types, including kidney (Burton et al., 2017), gastrointestinal (Gupta et al., 2009; Lv et al., 2014; Diemer et al., 2015), gingival (Cai et al., 2017), epidermal (Chanda et al., 2018), and amniotic stem cells (Russo et al., 2016); their evaluation with respiratory epithelial cells, however, has not been widely explored to date. Moreover, toxicity assessment was also warranted due to the use of solvents that have a potential risk of bearing residual toxicants as listed by ICH guidance. In tandem with comparisons of micro- and nanofibrous structure on epithelial behavior, the cells grew primarily as clusters with no evidence of fibroblastic morphology (Ravikrishnan et al., 2016). The regular presence of these clusters were likely due to hydrophobic and charged surface character of the PCL and chitosan, respectively, which might reduce or slow extensive monolayer formation. In the context of tracheal tissue engineering, PCL scaffolds tend to be investigated in blends as outlined in the introduction, though recent work by Mahoney and colleagues also found that PCL can support respiratory epithelial cell growth as a homo-polymer in addition to a co-polymer mix with chitosan (Mahoney et al., 2016). Similar to this study, our data also indicated PCL-based scaffolds were biocompatible with the Calu-3 bronchial epithelial cell line, irrespective of the absence or presence of chitosan. However, due to the reported potential for chitosan's additional bioactive effects on tracheal cell functionality (Risbud et al., 2001; Huang et al., 2009), along with putative antibacterial activity that could also be an advantage for preventing post-surgical infection (Hibbitts and O'Leary, 2018), PCL-Chitosan scaffolds were carried forward for atRA incorporation.

Retinoid molecules such as atRA exhibit diverse bioactivity on cell functionality, including cell proliferation and differentiation (Goncalves et al., 2019). Although known in the clinical field primarily for its role in the treatment of acute promyelocytic leukemia (Lo-Coco et al., 2013), atRA has been investigated in human trials as a therapeutic for pulmonary emphysema, albeit with mixed outcomes (Mao et al., 2002; Roth et al., 2006). In its pro-regenerative capacity, several preclinical studies have indicated that direct exposure to respiratory epithelium, as opposed to systemic drug delivery used in these trials, can provide significant benefit for epithelialization and resolution of lung injury (Gray et al., 2001; Huang et al., 2013). Indeed, our previous study with atRA-loaded collagen-based scaffolds showed that even a 6 h burst release of the drug was sufficient to influence primary respiratory epithelial cell mucociliary differentiation after 21 days of culture, without the need for repeat dosing (O'Leary et al., 2017). Therefore, in this study, one of our main aims was to design an atRA-loaded biomaterial platform with an augmented encapsulation efficiency through the use of a more hydrophobic polymer that complemented the molecules physicochemical properties (Pubchem RetinoicAcid, 2004). In this regard, the encapsulation efficiencies of atRA in the PCL-chitosan electrospun composites far surpassed the 11% encapsulation achieved with collagen-based materials (O'Leary et al., 2017). Following exposure of Calu-3 cells to these scaffolds with higher atRA loading, a non-significant reduction in cellular reduction of resazurin was detected, reflecting the growth suppressive effects that atRA is known to stimulate in other cancer cells (Lo-Coco et al., 2013). However, cells were ultimately viable in all sample groups, with no visible difference observed in the number of live and dead cells, negating concerns of atRA-mediated toxicity; rather, this reduction in cell metabolism could be possibly attributed to a shift in the airway cells from a more proliferative phenotype to the more differentiated mucociliary phenotype of the respiratory region, where cell turnover is slow under homeostatic physiological conditions (Stripp and Reynolds, 2008; Crosby and Waters, 2010).

While the atRA-loaded scaffolds successfully demonstrated their capacity as biocompatible substrates for respiratory epithelial growth, a clear improvement in the mucociliary phenotype of Calu-3 cells cultured on these scaffolds was not reproducibly attained, with different loading doses eliciting different responses. Mucin glycoprotein was expressed more in atRA-loaded biomaterials, although a statistically significant increase in transcription of the associated MUC5AC gene was not evident. This finding was in contrast to previous work with collagen-based biomaterials, where an approximate 51-fold increase was observed in primary tracheobronchial epithelial cells in monoculture (O'Leary et al., 2017). Similarly, while enhanced ciliation was observed in our previous work culturing primary cells on atRA-loaded collagen scaffolds, as reflected by a significant increase in FOXJ1 gene expression and apical localization of β-tubulin IV, no significant increases in FOXJ1 or evidence of motile cilia were seen in cells grown on atRA-loaded PCL-Chitosan scaffolds. There are several contributing factors in this study that could account for this. Firstly, although the Calu-3 cell line is considered as one that exhibits a tracheobronchial phenotype (Grainger et al., 2006), the cells might not have the same potential for ciliation as in primary cells. The presence of motile, elongated cilia of ≥7 μm is not consistently reported in the literature, and although we have previously utilized biomaterials to stimulate increased FOXJ1 expression (O'Leary et al., 2016), inherent differences between this cell line and primary respiratory epithelium are present (Pezzulo et al., 2011), which could contribute to the data in this study. Furthermore, previous studies in our laboratory had employed collagen-based biomaterials as the scaffold substrate, which is a natural material widely reported to enhance mucociliary behavior (Pfenninger et al., 2007; Fulcher et al., 2011). Of course, the presence of co-cultured mesenchymal cells such as fibroblasts or stromal cells can alter expression of these biomarkers (Le Visage et al., 2004; Kobayashi et al., 2007; O'Leary et al., 2016, 2017); while beyond the scope of this study, future work with primary epithelial and mesenchymal cells in co-culture would be the next step to evaluate scaffold-mediated regeneration of not only epithelium, but also submucosal tissue for structural integrity and vascularization (Chiang et al., 2016). Additionally, studies into the mechanical properties of the scaffold and potential adjustment of design features to improve epithelial differentiation are a point of interest, given the extensive research in the field of mechanobiology and cell-material mechanotransduction interactions [reviewed in (Almouemen et al., 2019)]. Both programs of future research could provide valuable information on the biomaterial's potential progression toward the clinic as an implant for tracheal trauma.

## Conclusion

In conclusion, this study has described the development and characterization of a novel, retinoic acid-releasing nanofibrous scaffold with potential for tracheal tissue regeneration. Advantageously, this scaffold integrates a synthetic polymer that has been used in human tracheal stents, a natural polymer generally regarded as safe (GRAS), and a drug with decades of use in patients. Coupled with the scalable nature of electrospinning as a fabrication method, all of these characteristics make the biomaterial outlined in this study amenable to translation as an implantable device for an unmet clinical need in tracheal replacement.

## Data Availability Statement

The datasets generated for this study are available on request to the corresponding author.

## Author Contributions

CO'L: project design, experimental work, personnel coordination, manuscript preparation and review. LS and AF-M: experimental work and manuscript review. II and BC: experimental work. FO'B: project design, personnel coordination, and manuscript review. S-AC: project lead, personnel coordination, and manuscript review.

### Conflict of Interest

The authors declare that the research was conducted in the absence of any commercial or financial relationships that could be construed as a potential conflict of interest.
