# Supplementary material for: The Fabrication and in vitro Evaluation of Retinoic Acid-Loaded Electrospun Composite Biomaterials for Tracheal Tissue Regeneration
Source: Front Bioeng Biotechnol. 2020 Mar 20;8:190. doi: 10.3389/fbioe.2020.00190 (PMC7103641; doi:10.3389/fbioe.2020.00190)
Supplement: Supplementary file 1 [file Table_1.pdf]

Supplementary Data Table 1: Polycaprolactone (PCL) nanofiber production. Results presented as mean diameter ± SD. n ≥ 31 fibers analyzed across a minimum of two scaffold batches.

**PCL Concentration (% w/v)**	**Needle Gauge**	**Flow Rate (µL/min)**	**Fiber Diameter (nm)**
7.5	18G	25<br>50<br>100	No fiber formation at all flow rates
10	18G	25<br>50<br>100	670 ± 515<br>1293 ± 1614<br>1450 ± 1310
20	18G	25<br>50<br>100	870 ± 600<br>1263 ± 1113<br>1733 ± 1445
30	18G	25<br>50<br>100	1623 ± 1498<br>1161 ± 1214<br>1953 ± 1731
40	18G	25<br>50<br>100	2374 ± 938<br>3647 ± 1386<br>2964 ±1682
50	18G	25<br>50<br>100	1822 ± 800<br>2392 ± 739<br>3833 ± 1324
